# Unveiling the Excited‐State Dynamics of Mn^2+^ in 0D Cs_4_PbCl_6_ Perovskite Nanocrystals

**DOI:** 10.1002/advs.202002210

**Published:** 2020-10-01

**Authors:** Wen Zhang, Jiaojiao Wei, Zhongliang Gong, Ping Huang, Jin Xu, Renfu Li, Shaohua Yu, Xingwen Cheng, Wei Zheng, Xueyuan Chen

**Affiliations:** ^1^ CAS Key Laboratory of Design and Assembly of Functional Nanostructures Fujian Key Laboratory of Nanomaterials State Key Laboratory of Structural Chemistry Fujian Institute of Research on the Structure of Matter, Chinese Academy of Sciences Fuzhou Fujian 350002 China; ^2^ College of Science North University of China Taiyuan Shanxi 030051 China; ^3^ Fujian Science and Technology Innovation Laboratory for Optoelectronic Information of China Fuzhou Fujian 350108 China

**Keywords:** energy transfer, excited‐state dynamics, manganese, perovskite nanocrystals, photoluminescence

## Abstract

Doping is an effective strategy for tailoring the optical properties of 0D Cs_4_PbX_6_ (X = Cl, Br, and I) perovskite nanocrystals (NCs) and expanding their applications. Herein, a unique approach is reported for the controlled synthesis of pure‐phase Mn^2+^‐doped Cs_4_PbCl_6_ perovskite NCs and the excited‐state dynamics of Mn^2+^ is unveiled through temperature‐dependent steady‐state and transient photoluminescence (PL) spectroscopy. Because of the spatially confined 0D structure of Cs_4_PbCl_6_ perovskite, the NCs exhibit drastically different PL properties of Mn^2+^ in comparison with their 3D CsPbCl_3_ analogues, including significantly improved PL quantum yield in solid form (25.8%), unusually long PL lifetime (26.2 ms), large exciton binding energy, strong electron–phonon coupling strength, and an anomalous temperature evolution of Mn^2+^‐PL decay from a dominant slow decay (in tens of ms scale) at 300 K to a fast decay (in 1 ms scale) at 10 K. These findings provide fundamental insights into the excited‐state dynamics of Mn^2+^ in 0D Cs_4_PbCl_6_ NCs, thus laying a foundation for future design of 0D perovskite NCs through metal ion doping toward versatile applications.

## Graphical abstract

The excited‐state dynamics of Mn^2+^ in 0D Cs_4_PbCl_6_ perovskite nanocrystals is unraveled through temperature‐dependent photoluminescence (PL) spectroscopy. Owing to the spatially confined 0D structure of Cs_4_PbCl_6_, the nanocrystals exhibit drastically different PL properties of Mn^2+^ in comparison with their 3D CsPbCl_3_ analogues, including significantly improved PL quantum yield in solid (25.8%) and unusually long PL lifetime (26.2 ms).

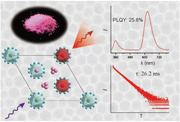

## 1 Introduction

0D perovskite derivative Cs_4_PbX_6_ (X = Cl, Br, and I) nanocrystals (NCs) have attracted increasing attention as a new class of optoelectronic materials owing to their excellent optical properties.^[^ ^1^ ^]^ These 0D perovskites, in particular Cs_4_PbBr_6_ with anomalous green photoluminescence (PL) coupled with remarkably improved quantum yield (QY) and long‐term stability over their 3D CsPbX_3_ cousins, have opened a new frontier in device engineering for light‐emitting diodes, lasers, and photodetectors, despite intense debate on the PL origin in this materials system.^[^ ^2^ ^]^ In contrast to 3D CsPbX_3_ perovskites with corner sharing [PbX_6_]^4−^ octahedra, the [PbX_6_]^4−^ units in 0D Cs_4_PbX_6_ are completely isolated from each other and surrounded by Cs^+^ cations.^[^ ^1c^ ^]^ As a result, these 0D perovskites show strong quantum confinement and exciton−phonon interactions, integrating the properties of both organic molecules and inorganic semiconductors, such as intrinsic Pb^2+^ ion emission, large exciton binding energy, and small polaron generation upon photoexcitation.^[^ ^3^ ^]^ Nonetheless, except for the green‐emitting Cs_4_PbBr_6_ with controversial origins, the Cs_4_PbX_6_ perovskites intrinsically do not luminesce in the visible region and have a wide bandgap,^[^ ^4^ ^]^ which limit their application as light‐emitting materials in a wide spectral range.

Metal ion doping is an effective way for fine‐tuning the electronic, optical, and magnetic properties of perovskite NCs.^[^ ^5^ ^]^ Recently, a diversity of metal ions such as transition‐metal and rare‐earth ions have been incorporated into perovskite NCs to enhance their PL efficiency and stability and to produce new emission bands by taking advantage of the energy transfer from the excitons to the dopants.^[^ ^6^ ^]^ Among diverse metal ions, Mn^2+^ ions have been recognized as one of the most effective dopants for perovskite NCs, due to very efficient exciton‐to‐Mn^2+^ energy transfer which brings about many intriguing optical properties, such as dominant Mn^2+^ emission and significantly improved PLQY and stability.^[^ ^7^ ^]^ Specifically, Mn^2+^ ions have been exploited as spectroscopic probes in Cs_4_PbX_6_ NCs to stabilize the 0D perovskite structure and probe the optical behaviors of molecule‐like isolated octahedra.^[^ ^8^ ^]^ However, the coexistence of 3D CsPbX_3_ impurity in 0D Cs_4_PbX_6_ perovskites may influence seriously the spectroscopic analyses and lead to an unreliable conclusion. Thus far, the PL properties of Mn^2+^ in pure‐phase Cs_4_PbX_6_ perovskites including the exciton‐to‐Mn^2+^ energy transfer and excited‐state dynamics of Mn^2+^, which are of fundamental importance for tailoring the optical properties of 0D perovskites and expanding their applications, remain unexplored.

Herein, we develop a unique strategy for the controlled synthesis of Mn^2+^‐doped 0D Cs_4_PbCl_6_ perovskite NCs via a modified hot‐injection method. The as‐synthesized NCs are monodispersed, highly crystallized, and pure phase free of CsPbCl_3_ impurity, which enable an accurate elaboration of the PL properties of Mn^2+^ in Cs_4_PbCl_6_ NCs. The effect of Mn^2+^ doping concentrations on the electronic structure and optical properties of the NCs as well as the efficiency of energy transfer from the excitons to Mn^2+^ are systematically investigated through PL and PL decay measurements. Furthermore, by means of temperature‐dependent steady‐state/transient PL and electron paramagnetic resonance (EPR) spectroscopies, the activation energy, electron−phonon coupling strength, and excited‐state dynamics of Mn^2+^ in Cs_4_PbCl_6_ NCs are unveiled.

## 2 Results and Discussion

**Figure 1:**
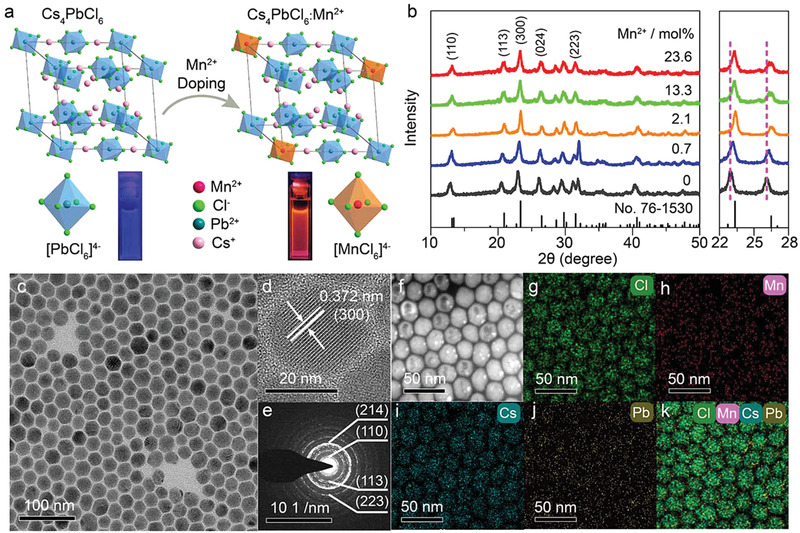
a) Crystal structure of rhombohedral Cs_4_PbCl_6_ and the crystallographic site for Mn^2+^ dopants. The PL photographs of Cs_4_PbCl_6_ NCs dispersed in cyclohexane under 304 nm ultraviolet lamp irradiation are presented, showing a PL color change from blue to orange‐red upon Mn^2+^ doping. b) XRD patterns of Cs_4_PbCl_6_:Mn^2+^ NCs with different Mn^2+^ doping concentrations. The bottom lines represent the standard XRD pattern of rhombohedral Cs_4_PbCl_6_ (JCPDS No. 76‐1530). The enlarged 2*θ* range (22°–28°) of XRD patterns shows a monotonic shift of the diffraction peaks with increasing the Mn^2+^ concentration. c) TEM image, d) HRTEM image, e) SAED pattern, f) STEM image, and g–k) the corresponding elemental mappings (Cs, Pb, Mn, and Cl) of Cs_4_PbCl_6_:23.6% Mn^2+^ NCs.

The Cs_4_PbCl_6_ crystal has a perovskite‐derivative 0D structure (space group *R* $\bar{3}$ *c*) with isolated [PbCl_6_]^4−^ octahedra surrounded by Cs^+^ cations. Mn^2+^ dopants are supposed to substitute the octahedral Pb^2+^ site (**Figure** **1**a). High‐quality Mn^2+^‐doped Cs_4_PbCl_6_ NCs were synthesized through a modified hot‐injection method by using benzoyl chloride as the halide source to precipitate the NCs.^[^ ^9^ ^]^ The Mn^2+^ doping concentrations were controlled by varying the molar ratio of Pb to Mn in the precursor solution. The chemical compositions of the NCs were checked by energy dispersive X‐ray spectroscopy (EDS) and the actual Mn^2+^ doping levels were identified by inductively coupled plasma‐atomic emission spectroscopy (ICP‐AES), showing a variation of Mn^2+^ from 0.7 to 23.6 mol% (Table S1, Supporting Information). Powder X‐ray diffraction (XRD) measurements show that all diffraction peaks of the NCs can be well indexed into rhombohedral Cs_4_PbCl_6_ (Joint Committee on Powder Diffraction Standards (JCPDS) No. 76‐1530) without any observable impurities (Figure 1b). The diffraction peaks (e.g., at 22.9° and 26.1°) of the NCs shift to higher angles with increasing the Mn^2+^ concentration, as a result of lattice contraction induced by the substitution of Pb^2+^ (0.133 nm) with smaller Mn^2+^ (0.097 nm).^[^ ^7a^ ^]^ It should be noted that further elevating the Mn^2+^ concentration will result in impurities of CsCl and CsMnCl_3_ due to spinodal decomposition of the NCs (Figure S1, Supporting Information).^[^ ^10^ ^]^ Transmission electron microscopy (TEM) images show that the NCs have a hexagonal morphology with mean sizes ranging from 23.6 ± 1.9 to 25.9 ± 2.1 nm (Figure 1c and Figure S2, Supporting Information), indicating that the doping of Mn^2+^ with nominal concentrations below 30 mol% has little influence on the morphologies and sizes of the resulting NCs. High‐resolution TEM (HRTEM) images (Figure 1d and Figure S3, Supporting Information) display clear lattice fringes with observed d spacings of 0.380 and 0.372 nm, respectively, for the (300) plane of the undoped and 23.6 mol% Mn^2+^‐doped Cs_4_PbCl_6_ NCs, confirming lattice contraction of the NCs induced by Mn^2+^ doping. The pure phase and high crystallinity of the resulting NCs were further verified by their clearly indexed selected‐area electron diffraction (SAED) ring pattern (Figure 1e). Elemental mappings by scanning transmission electron microscopy (STEM) (Figure 1f–k) and X‐ray photoelectron spectroscopy (XPS) (Figure S4, Supporting Information) revealed the successful doping of Mn^2+^ into the lattice of Cs_4_PbCl_6_ NCs.

**Figure 2:**
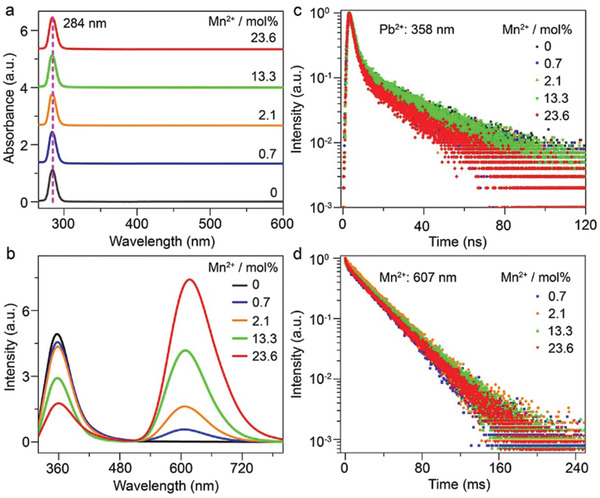
a) Optical absorption spectra and b) PL emission spectra (*λ*_ex_ = 289 nm) of Cs_4_PbCl_6_:Mn^2+^ NCs with different Mn^2+^ doping concentrations. PL decays from c) ^3^P_1_ level of Pb^2+^ and d) ^4^T_1g_ level of Mn^2+^ in Cs_4_PbCl_6_:Mn^2+^ NCs with different Mn^2+^ doping concentrations by monitoring the Pb^2+^ and Mn^2+^ emissions at 358 and 617 nm, respectively. All the spectra were recorded at room temperature.

**Figure** **2**a and Figure S5 in the Supporting Information show the optical absorption spectra of Cs_4_PbCl_6_:*x* mol% Mn^2+^ (*x* = 0, 0.7, 2.1, 13.3, and 23.6) NCs. All the NCs displayed an intense and narrow absorption band (full width at half maximum (FWHM) ≈172 meV) at 284 nm (4.37 eV), which is independent of Mn^2+^ doping concentration and is attributed to the localized exciton absorption of Cs_4_PbCl_6_ originating from the spin‐orbital allowed ^1^S_0_ (^1^A_g_) → ^3^P_1_ (^3^T_1u_) transition of Pb^2+^ in isolated [PbCl_6_]^4−^ octahedra.^[^ ^11^ ^]^ The essentially identical exciton absorption band in Mn^2+^‐doped Cs_4_PbCl_6_ NCs with varying Mn^2+^ concentrations suggests that Mn^2+^ doping has little influence on the electronic structure of Cs_4_PbCl_6_ host; this differs from that of Mn^2+^‐doped CsPbCl_3_ where Mn^2+^ doping resulted in a blueshift in the exciton absorption band edge due to the effect of Mn^2+^ alloying on the bandgap of CsPbCl_3_.^[^ ^7a^ ^]^ No absorption tail was observed in the spectral range from 350 to 450 nm, affirming that the NCs were free of CsPbCl_3_ impurity.

Upon excitation at 289 nm, all the NCs exhibited a broad emission band (FWHM ≈ 554 meV) at 358 nm; while in Mn^2+^‐doped NCs, an additional emission band (FWHM ≈ 310 meV) around 617 nm was also observed (Figure 2b). The emission band at 358 nm agreed well with that of Pb^2+^ emission in Cs_4_PbCl_6_ perovskites previously reported and can be assigned to the electronic transitions of Pb^2+^ from ^3^P_0,1_ levels to ^1^S_0_ level.^[^ ^8^, ^11^ ^]^ The emission band at 617 nm was ascribed to the spin‐forbidden ^4^T_1g_ → ^6^A_1g_ transition of Mn^2+^ occupying the octahedral Pb^2+^ sites, as well documented in Mn^2+^‐doped CsPbCl_3_ NCs.^[^ ^7a–c^ ^]^ The PL intensity of Mn^2+^ increased gradually at the expense of that of Pb^2+^ with increasing the Mn^2+^ concentration, indicative of an efficient energy transfer from Pb^2+^ to Mn^2+^ in Cs_4_PbCl_6_:Mn^2+^ NCs,^[^ ^8^ ^]^ as also evidenced by the nearly same exciton excitation band of Pb^2+^ at 289 nm for the Pb^2+^ and Mn^2+^ emissions at 358 and 617 nm, respectively (Figure S6, Supporting Information). Coincidentally, we found that the overall PL intensity of the NCs was enhanced by factors of 1.2, 1.5, 1.9, and 3.1 as the Mn^2+^ concentration increased from 0 to 0.7, 2.1, 13.3, and 23.6 mol%, respectively (Figure S7, Supporting Information), as a merit of efficient energy transfer from Pb^2+^ to Mn^2+^ which alleviates the energy migration through Pb^2+^ sublattice to the intrinsic or surface defects of the NCs.^[^ ^12^ ^]^ Because the [PbX_6_]^4−^ and [MnCl_6_]^4−^ octahedra in 0D Cs_4_PbCl_6_:Mn^2+^ perovskite are completely isolated from each other and electronically decoupled, the Pb^2+^‐Mn^2+^ or Mn^2+^‐Mn^2+^ exchange interactions are inhibited in Cs_4_PbCl_6_:Mn^2+^ NCs.^[^ ^3^, ^4^ ^]^ As a result, the Pb^2+^‐to‐Mn^2+^ energy transfer in Cs_4_PbCl_6_:Mn^2+^ NCs cannot be induced by an exchange interaction; instead, it is most probably dictated by an electric multipole interaction between Pb^2+^ and Mn^2+^, as previously reported in Pb^2+^/Mn^2+^ codoped NaCl and KCl crystals.^[^ ^13^ ^]^ Such Pb^2+^‐to‐Mn^2+^ energy transfer was further corroborated by the decreased PL lifetime of Pb^2+^ with increasing the Mn^2+^ concentration (Figure 2c). From the PL lifetimes of Pb^2+^, the highest efficiency of energy transfer from Pb^2+^ to Mn^2+^ was calculated to be 46.0%, in 23.6 mol% Mn^2+^‐doped NCs (Table S2, Supporting Information). Intriguingly, we observed that the PL intensity of Mn^2+^ in NCs of different Mn^2+^ concentrations exhibited a nearly identically single‐exponential decay with a fitted PL lifetime of ≈26.2 ms (Figure 2d and Table S2, Supporting Information). Such a long Mn^2+^‐PL lifetime in Cs_4_PbCl_6_:Mn^2+^ NCs is much longer than the typical Mn^2+^‐PL lifetime (in the order of magnitude of 1 ms) in CsPbCl_3_:Mn^2+^ NCs where the exciton‐to‐Mn^2+^ energy transfer was dictated by an exchange interaction.^[^ ^7d–f^ ^]^ The nearly identical PL decay dynamics of Mn^2+^ in Cs_4_PbCl_6_:Mn^2+^ NCs of different Mn^2+^ concentrations is due to the inhibition of exchange interaction between Mn^2+^ ions in the structurally confined [MnCl_6_]^4−^ octahedra and the single exponential decay indicates a homogeneous distribution of Mn^2+^ at the octahedral Pb^2+^ site.^[^ ^8^ ^]^ The absolute PLQYs of Mn^2+^ were determined to be 7.1, 20.0, 31.8, and 54.2% in NC solution, and 3.5, 9.9, 18.0, and 25.8% in NC powder, with Mn^2+^ doping concentrations of 0.7, 2.1, 13.3, and 23.6 mol%, respectively (Table S3 and Figure S8, Supporting Information). It is worth mentioning that the obtained PLQYs of Cs_4_PbCl_6_:Mn^2+^ powder are much higher than those (≈0.1%) of CsPbX_3_ counterparts previously reported.^[^ ^1a^ ^]^ Such superior PLQYs of Cs_4_PbCl_6_:Mn^2+^ NC solid are attributed to the efficient exciton‐to‐Mn^2+^ energy transfer and the spatially confined 0D structure of Cs_4_PbCl_6_ perovskite that favors high exciton binding energy.^[^ ^8^ ^]^

**Figure 3:**
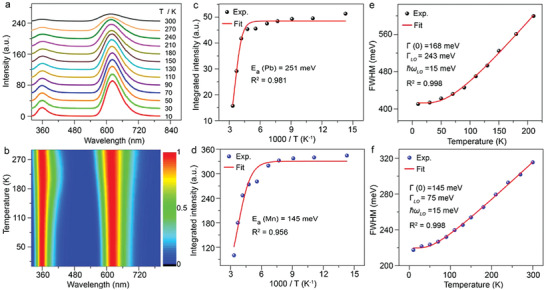
a) Temperature‐dependent PL emission spectra (*λ*_ex_ = 289 nm) and b) the corresponding wavelength‐temperature contour plot of Cs_4_PbCl_6_:23.6% Mn^2+^ NCs. Integrated PL intensities and FWHMs of c,e) Pb^2+^ and d,f) Mn^2+^ as a function of temperature. The activation energy (*E*_a_) and the electron–phonon coupling strength (Γ_LO_) of Pb^2+^ and Mn^2+^ in Cs_4_PbCl_6_:Mn^2+^ NCs as well as the LO phonon energy (ℏ*ω*_LO_) of the NC lattice were derived by fitting to the data in (c)–(f).

To gain deep insights into the excited‐state dynamics of Mn^2+^ in Cs_4_PbCl_6_ NCs, we carried out temperature‐dependent steady‐state and transient PL spectroscopic measurements. **Figure** **3**a,b shows the temperature‐dependent PL emission spectra of Cs_4_PbCl_6_:23.6% Mn^2+^ NCs in the temperature range of 10–300 K upon excitation at 289 nm. As the temperature fell from 300 to 10 K, the PL intensities of Pb^2+^ and Mn^2+^ increased significantly by factors of 3.5 and 3.6, with their emission bandwidth narrowing from 574 and 315 meV to 410 and 218 meV, respectively (Figure 3b). The increase in PL intensities of Pb^2+^ and Mn^2+^ and the spectral narrowing with the temperature decrease can be attributed, respectively, to the smaller nonradiative transition probability and reduced electron–phonon coupling of Pb^2+^ and Mn^2+^ at lower temperatures.^[^ ^14^ ^]^ Notably, the PL intensity ratio of Mn^2+^ to Pb^2+^ remained essentially unchanged with the temperature decrease (Figure S9, Supporting Information), indicating that the Pb^2+^‐to‐Mn^2+^ energy transfer in Cs_4_PbCl_6_:Mn^2+^ NCs is temperature independent. This is drastically different from that of Mn^2+^‐doped CsPbCl_3_ where the PL intensity ratio of Mn^2+^ to exciton decreased remarkably with the temperature decrease due to the reduced exciton‐to‐Mn^2+^ energy transfer at lower temperatures.^[^ ^15^ ^]^ Besides, a slight redshift in the emission band of Mn^2+^ from 615 to 624 nm was observed when the temperature fell from 300 to 10 K, as a result of lattice contraction which led to larger d–d splitting of Mn^2+^ ions and correspondingly lowered the energy gap of the ^4^T_1g_ → ^6^A_1g_ transition.^[^ ^15a^ ^]^ It is worth mentioning that the lattice contraction at low temperatures may cause a PL blueshift in conventional semiconductors such as CdSe and GaAs,^[^ ^16^ ^]^ while it usually induced a PL redshift in halide perovskites such as CsPbBr_3_ NCs, as a result of the opposite effects of thermal expansion and electron–phonon interaction on the bandgap energy.^[^ ^17^ ^]^ Figure 3c,d depicts the integrated PL intensities of Pb^2+^ and Mn^2+^ as a function of inverse temperature^[^ ^18^ ^]^

(1) $$\begin{array}{c} I\left(T\right)=\frac{I_{0}}{1+Ae^{-E_{a}/\left(k_{B}T\right)}} \end{array}$$

where *I*(*T*) and *I*_0_ are the integrated PL intensities at *T* and 0 K, respectively, *E*_a_ is the activation energy, and *k*_B_ is the Boltzmann constant. The activation (or binding) energies of Pb^2+^ and Mn^2+^ were determined to be 251 and 145 meV, respectively, which are much larger than the exciton binding energy (55–75 meV) of CsPbCl_3_ NCs, revealing the typical Frenkel‐type excitons in Cs_4_PbCl_6_:Mn^2+^ NCs.^[^ ^19^ ^]^ To extract the electron–phonon coupling strength, we fitted the temperature dependence of the FWHMs of Pb^2+^ and Mn^2+^ emissions using the equation (Figure 3e,f)^[^ ^20^ ^]^

(2) $$\begin{array}{c} \Gamma \left(T\right)=\Gamma \left(0\right)+\sigma T+\frac{\Gamma_{\mathrm{LO}}}{e^{\hbar \omega_{\mathrm{LO}}/k_{B}T}-1} \end{array}$$

where *Γ*(*T*) is the FWHM at temperature *T*, *Γ*(0) is the inhomogeneous broadening, *σ* denotes the electron–acoustic phonon coupling which is usually negligible and set to 0 for fitting, ℏ*ω*_LO_ is the longitudinal optical (LO) phonon energy, and *Γ*_LO_ is the electron–LO phonon coupling strength. The electron–phonon coupling strength (*Γ*_LO_) of Pb^2+^ and Mn^2+^ were determined to be 243 and 75 meV, respectively, and the LO phonon energy (ℏ*ω*_LO_) was fitted to be 15 meV, which is consistent with that determined by Raman spectra reported previously.^[^ ^21^ ^]^ Such high exciton binding energy and strong electron–phonon coupling strength of Mn^2+^ in Cs_4_PbCl_6_:Mn^2+^ NCs resulted in the superior PLQYs of the NC solid to their CsPbCl_3_:Mn^2+^ analogues, making them promising luminescent materials for efficient solid‐state lighting applications.

**Figure 4:**
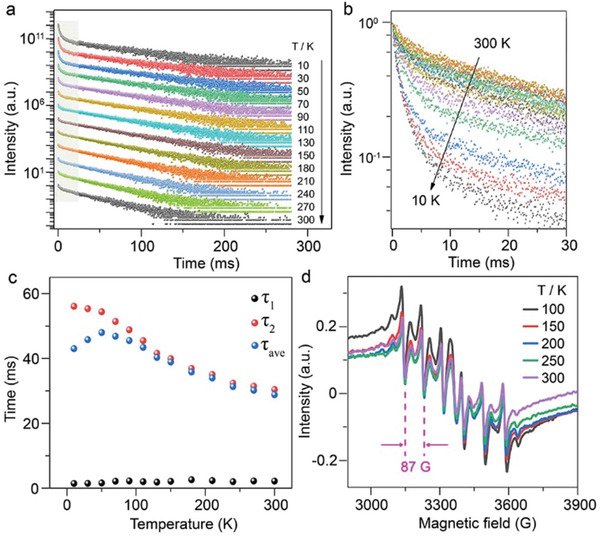
a) Temperature‐dependent PL decay curves (*λ*_ex_ = 289 nm) of Cs_4_PbCl_6_:23.6% Mn^2+^ NCs by monitoring the Mn^2+^ emission at its maximum around 617 nm. The decay curves were stacked up to make each of them more visible. The initial fast decay portion marked by shadow was enlarged in (b), showing a remarkable increase in the amplitude of the fast Mn^2+^‐PL decay with the temperature decrease. c) The time constants of the fast decay component (*τ*_1_), the slow decay component (*τ*_2_), and the average lifetimes (*τ*_ave_) of Mn^2+^ as a function of temperature, determined by biexponential fitting to the decay curves in (a). d) X‐band EPR spectra of Mn^2+^ in Cs_4_PbCl_6_:23.6% Mn^2+^ NCs measured at different temperatures.

**Figure** **4**a and Figure S10 in the Supporting Information show the temperature‐dependent PL decay curves of Cs_4_PbCl_6_:23.6% Mn^2+^ NCs in the temperature range from 10 to 300 K by monitoring the Mn^2+^ emission at its maximum around 617 nm and Figure 4b enlarges the initial fast decay portion of the decay curves. All the decay curves can be well fitted to a biexponential function $I\;\left(t\right)=A_{1}\;e^{-t/\tau_{1}}+A_{2}e^{-t/\tau_{2}}$ consisting of a fast decay with a time constant *τ*_1_ around 1–2 ms and a slow decay with a time constant *τ*_2_ in tens of ms scale. The average lifetimes were determined by the expression $\tau_{\mathrm{ave}}=\left(A_{1}\;\tau_{1}^{2}+A_{2}\tau_{2}^{2}\right)/\left(A_{1}\tau_{1}+A_{2}\tau_{2}\right)$. The time constants and the amplitudes of the decay components were summarized in Table S4 in the Supporting Information. The slow Mn^2+^‐PL decay was ascribed to the electronic transition of Mn^2+^ in isolated [MnCl_6_]^4−^ octahedra, and its lifetime (*τ*_2_) increased gradually from 30.4 to 56.1 ms as the temperature fell from 300 to 10 K (Figure 4c), due to the reduction of nonradiative transition probability.^[^ ^3^, ^14^ ^]^ Specifically, we found that the amplitude of the fast Mn^2+^‐PL decay in Cs_4_PbCl_6_:Mn^2+^ NCs increased considerably with the temperature decrease and became dominant when the temperature fell below 70 K (Figure 4b). Such anomalous temperature evolution of Mn^2+^‐PL decay is independent of Mn^2+^ doping concentration, as also observed in NCs with a low Mn^2+^ doping level of 0.7 mol% (Figure S11, Supporting Information). This can be well interpreted that the high‐symmetry [MnCl_6_]^4−^ octahedra in Cs_4_PbCl_6_:Mn^2+^ NCs may gradually break down into low‐symmetry one because of low‐temperature‐induced lattice contraction,^[^ ^22^ ^]^ which led to the increase of radiative transition probability and the introduction of an additional nonradiative pathway stemmed from strong Mn–Mn dipole–dipole coupling interactions, as recently reported by Wu and co‐workers in [Mn_4_In_16_S_35_] nanoclusters.^[^ ^23^ ^]^ Note that the origin of the fast Mn^2+^‐PL decay from CsPbCl_3_:Mn^2+^ impurity can be explicitly ruled out, as further confirmed by the absence of CsPbCl_3_ absorption for Mn^2+^ emission in the excitation spectra of the NCs at low temperatures (Figure S12, Supporting Information). The structural integrity of the NCs and the enhanced Mn–Mn dipole–dipole interactions at low temperatures can to some extent be evidenced by the slight broadening of the EPR bands of Mn^2+^ as the temperature fell from 300 to 100 K (Figure 4d and Figure S13, Supporting Information). The EPR spectra of the NCs exhibited sharp sextet hyperfine splitting patterns of isolated Mn^2+^ ions with a hyperfine constant *A* = 87 G, confirming the substitution of Mn^2+^ in octahedral Pb^2+^ sites in 0D Cs_4_PbCl_6_ NCs.^[^ ^3^, ^7^ ^]^ These results demonstrate that the excited‐state dynamics of Mn^2+^ is sensitive to the surrounding crystal‐field environment, revealing the great potential of Mn^2+^ ions as sensitive spectroscopic and structural probes in 0D perovskites for probing the molecule‐like isolated octahedra.

## 3 Conclusions

In summary, we have systematically investigated the excited‐stated dynamics of Mn^2+^ in pure‐phase 0D Cs_4_PbCl_6_ perovskite NCs through temperature‐dependent steady‐state and transient PL spectroscopy. The substitution of Pb^2+^ by Mn^2+^ in isolated [PbCl_6_]^4−^ octahedra resulted in intense Mn^2+^ emission through efficient Pb^2+^‐to‐Mn^2+^ energy transfer. Owing to the spatially confined 0D structure of Cs_4_PbCl_6_ perovskite, Mn^2+^ ions in these NCs exhibited a large binding energy (145 meV) and strong electron–phonon coupling strength (75 meV), which led to a remarkably long PL lifetime (26.2 ms) of Mn^2+^ and a superior PLQY (25.8%) for NC solids to their 3D CsPbCl_3_ analogues. Specifically, an anomalous temperature evolution of Mn^2+^‐PL decay from a dominant slow decay with a time constant of ≈30.4 ms at 300 K to a fast decay with a time constant of ≈1.47 ms at 10 K was observed, as a result of strong Mn–Mn dipole–dipole coupling interactions induced by lattice contraction at low temperatures. The unambiguous revelation of the excited‐state dynamics of Mn^2+^ in pure‐phase Cs_4_PbCl_6_ NCs is of vital importance for future design and development of 0D perovskite NCs toward versatile applications such as solid‐state lighting.

## 4 Experimental Section

##### Chemicals and Materials

Pb(CH_3_COO)_2_·3H_2_O (99.99%), Mn(CH_3_COO)_2_·4H_2_O (99.99%), Cs_2_CO_3_ (99.9%), and benzoyl chloride (C_7_H_5_ClO, 98.0%) were purchased from Aladdin (Shanghai, China). Oleic acid (OA, 90%), oleylamine (OAm, 90%), and 1‐octadecene (ODE, 90%) were purchased from Sigma‐Aldrich (Shanghai, China). Cyclohexane (analytical grade) was purchased from Sinopharm Chemical Reagent Co. (Shanghai, China). All chemicals were used as received without further purification.

##### Synthesis of Cs_4_PbCl_6_:Mn^2+^ NCs

High‐quality Cs_4_PbCl_6_:Mn^2+^ NCs were synthesized through a modified hot‐injection method by using benzoyl chloride as the halide source to precipitate the NCs. In a typical synthesis of Cs_4_PbCl_6_:30 mol% Mn^2+^ (nominal concentration) NCs, 0.28 mmol of Pb(CH_3_COO)_2_·3H_2_O, 0.12 mmol of Mn(CH_3_COO)_2_·4H_2_O, and 0.8 mmol of Cs_2_CO_3_ were added to a 100 mL two‐neck flask containing 3 mL of OA, 4 mL of OAm, and 13 mL of ODE. The resulting mixture was then heated to 140 °C under a N_2_ flow with constant stirring for 1 h to remove the moisture from the raw materials and dissolve the powder. After the solution become clear, the temperature was raised up to 180 °C and stabilized for 10 min, followed by rapid injection of 2.4 mmol of benzoyl chloride into the hot solution. After 5 min of reaction, the mixture was cooled down to room temperature by ice‐water bath. The NCs were precipitated by centrifugation of the mixture at 4000 rpm for 5 min, washed with 2 mL of cyclohexane and collected by centrifugation again at 12 000 rpm for 5 min. Finally, half of the precipitate was redispersed in 10 mL of cyclohexane and the other half was dried in the oven to make into powder. For the synthesis of NCs with different Mn^2+^ doping concentrations, different molar ratio of Pb to Mn with 0.4 mmol of total amount of (Pb + Mn) in the precursor solution was used under otherwise identical conditions. The nominal Mn^2+^ doping concentration was defined by the molar ratio of Mn to (Pb + Mn) in the precursor solution, and the actual Mn^2+^ doping concentrations were identified by ICP‐AES.

##### Structural and Optical Characterization

Powder XRD patterns of the samples were collected with an X‐ray diffractometer (MiniFlex2, Rigaku) using Cu *K*_*α*1_ radiation (*λ* = 0.154 187 nm). Both the low‐ and high‐resolution TEM measurements were performed by using a TECNAI G^2^ F20 TEM. The STEM image for Cs_4_PbCl_6_:Mn^2+^ NCs coupled with the EDS elemental mapping was obtained by utilizing a Titan G^2^ 80‐200 ChemiSTEM, FEI. ICP analysis was conducted on an ICP‐AES spectrometer (Ultima2, Jobin Yvon). XPS measurements were carried out on a Thermo Fisher ESCALAB 250Xi using Al *K*_*α*_ (1486.6 eV) and He *I*_*α*_ (21.2 eV) as the sources of radiation. X‐band EPR spectra were recorded on a Bruker ER‐420 spectrometer with a 100 KHz magnetic field in X‐band and an electronic field of 9655.448 MHz. Optical absorption spectra of the NCs were collected with a Perkin‐Elmer Lambda365 UV/Vis spectrometer in transmission mode. 2 mL of cyclohexane solution (100 µg mL^−1^) of the NCs was used for measurement and 2 mL of pure cyclohexane was used as the reference. PL excitation and emission spectra and PL decays were recorded on the FLS980 spectrometer (Edinburgh) equipped with both continuous (450 W) and pulsed xenon lamp. PL photographs of the NC solution were taken by using a Huawei P30Pro without using any filter. The absolute PLQYs of the samples were measured by employing a standard barium sulfate coated integrating sphere (150 mm in diameter, Edinburgh) as the sample chamber that was mounted on the FLS980 spectrometer with the entry and output port of the sphere located in 90° geometry from each other in the plane of the spectrometer. A standard tungsten lamp was used to correct the optical response of the instrument. For low temperature measurement, samples were mounted on a closed cycle cryostat (10–350 K, DE202, Advanced Research Systems). All the spectral data were recorded at room temperature based on the NC powder samples unless otherwise noted and corrected for the spectral response of both the spectrometer and the integrating sphere.

## Conflict of Interest

The authors declare no conflict of interest.

## Supporting information

Supporting Information
