# Supplementary material for: Unveiling the Excited‐State Dynamics of Mn2+ in 0D Cs4PbCl6 Perovskite Nanocrystals
Source: Adv Sci (Weinh). 2020 Oct 1;7(22):2002210. doi: 10.1002/advs.202002210 (PMC7675042; doi:10.1002/advs.202002210)
Supplement: Supplementary file 1 — Supporting Information [file ADVS-7-2002210-s001.pdf]

© 2020 Wiley-VCH GmbH

ADVANCED SCIENCE
Open Access

Supporting Information

for *Adv. Sci.,* DOI: 10.1002/advs.202002210

Unveiling the Excited-State Dynamics of Mn^{2+} in 0D Cs_4PbCl_6 Perovskite Nanocrystals

Wen Zhang, Jiaojiao Wei, Zhongliang Gong, Ping Huang, Jin Xu, Renfu Li, Shaohua Yu, Xingwen Cheng, Wei Zheng, and Xueyuan Chen**

Copyright WILEY-VCH Verlag GmbH & Co. KGaA, 69469 Weinheim, Germany, 2016.

Supporting Information

Unveiling the excited-state dynamics of Mn^{2+} in zero-dimensional Cs_4PbCl_6 perovskite nanocrystals

Wen Zhang[abc], Jiaojiao Wei[a], Zhongliang Gong[a], Ping Huang[ac], Jin Xu[ac], Renfu Li[ac], Shaohua Yu[a], Xingwen Cheng[a], Wei Zheng[ac], and Xueyuan Chen[ac]**

[a]CAS Key Laboratory of Design and Assembly of Functional Nanostructures, Fujian Key Laboratory of Nanomaterials, and State Key Laboratory of Structural Chemistry, Fujian Institute of Research on the Structure of Matter, Chinese Academy of Sciences, Fuzhou, Fujian 350002, China.

Fax: +86-591-63179421; Tel: +86-591-63179421;

E-mail: zhengwei@fjirsm.ac.cn; xchen@fjirsm.ac.cn

[b]College of Science, North University of China, Taiyuan, Shanxi 030051, China.

[c]Fujian Science & Technology Innovation Laboratory for Optoelectronic Information of China, Fuzhou, Fujian 350108, China.

Table S1. Nominal and actual Mn^{2+} doping concentrations in Cs_4PbCl_6:Mn^{2+} NCs synthesized with different molar ratio of Pb to Mn in the precursor solution. The nominal Mn^{2+} doping concentration was defined by the molar ratio of Mn to (Pb + Mn) in the precursor solution, and the actual Mn^{2+} doping concentrations were identified by inductively coupled plasma-atomic emission spectroscopy (ICP-AES).

Sample	Pb : Mn	Nominal / mol%	Actual / mol%
1	1 : 0	0	0
2	19 : 1	5	0.7
3	9 : 1	10	2.1
4	4 : 1	20	13.3
5	7 : 3	30	23.6

Table S2. PL lifetimes of Pb^{2+} and Mn^{2+} and the efficiency (η_{ET}) of energy transfer from Pb^{2+} to Mn^{2+} in Cs_4PbCl_6:Mn^{2+} NCs of different Mn^{2+} doping concentrations. The PL lifetimes of Mn^{2+} were obtained by single-exponential fitting to the decay curves of Mn^{2+}. The PL decay curves of Pb^{2+} were fitted to a biexponential function $\mathrm{I(t)} = A_1e^{-t/\tau_1} + A_2e^{-t/\tau_2}$, and the time constants and the amplitudes of the decay components were summarized in the table. The average lifetimes of Pb^{2+} were determined by $\tau_{ave} = (A_1\tau_1^2 + A_2\tau_2^2)/(A_1\tau_1 + A_2\tau_2)$. The Pb^{2+}-to-Mn^{2+} energy transfer efficiency (η_{ET}) was calculated by $\eta_{ET} = \frac{\tau_{ave}^0 - \tau_{ave}^x}{\tau_{ave}^0} \times 100\%$, where τ_{ave}^0 and τ_{ave}^x represent the average PL lifetimes of Pb^{2+} in Cs_4PbCl_6:Mn^{2+} NCs with Mn^{2+} doping concentration of 0 and x mol%, respectively.

x mol% Mn^{2+}		0	0.7	2.1	13.3	23.6
Mn^{2+}	τ / ms	–	26.1	26.4	27.0	26.2
Pb^{2+}	τ_1 / ns	2.31	2.35	2.27	2.31	2.18
	A_1	0.40	0.17	0.37	1.14	1.12
	τ_2 / ns	28.0	24.2	25.7	26.0	21.6
	A_2	0.12	0.12	0.12	0.15	0.12
	τ_{ave} / ns	22.5	21.4	20.8	16.5	12.2
η_{ET} / %		–	4.9	7.6	26.7	46.0

Table S3. PLQYs for Pb^{2+} and Mn^{2+} emissions and the overall PLQYs of Cs_4PbCl_6:Mn^{2+} NCs with different Mn^{2+} doping concentrations. Both the NC solution (2 mg mL^{-1} in cyclohexane) and NC powder samples were used for PLQY measurement.

x mol%	NC solution			NC powder		
Mn^{2+}	Pb^{2+}	Mn^{2+}	Overall	Pb^{2+}	Mn^{2+}	Overall
0	5.9	–	5.9	3.7	–	3.7
0.7	5.7	7.1	12.8	0.5	3.5	4.0
2.1	5.3	20.0	25.3	0.4	9.9	10.3
13.3	4.8	31.8	36.6	0.2	18.0	18.2
23.6	3.0	54.2	57.2	0.1	25.8	25.9

Table S4. Time constants, amplitudes of the decay components, and average PL lifetimes of Mn^{2+} obtained by biexponential fitting to the decay curves of Mn^{2+} in Cs_4PbCl_6:23.6% Mn^{2+} NCs at different temperatures. The average lifetimes of Mn^{2+} were determined by $\tau_{ave} = (A_1\tau_1^2 + A_2\tau_2^2)/(A_1\tau_1 + A_2\tau_2)$.

Temperature / K	τ_1 / ms	A_1	τ_2 / ms	A_2	τ_{ave} / ms
10	1.47	0.821	56.1	0.069	43.0
30	1.48	0.771	55.3	0.096	45.8
50	1.59	0.649	54.4	0.138	48.0
70	2.17	0.576	51.4	0.239	46.9
90	2.25	0.532	48.8	0.322	45.5
110	2.02	0.473	45.5	0.417	43.4
130	1.86	0.383	41.6	0.515	40.3
150	2.12	0.306	40.0	0.572	38.9
180	2.62	0.306	36.9	0.658	35.8
210	2.35	0.331	35.1	0.659	34.0
240	2.00	0.324	32.4	0.565	31.3
270	2.24	0.366	31.4	0.588	30.2
300	2.21	0.408	30.4	0.497	28.8

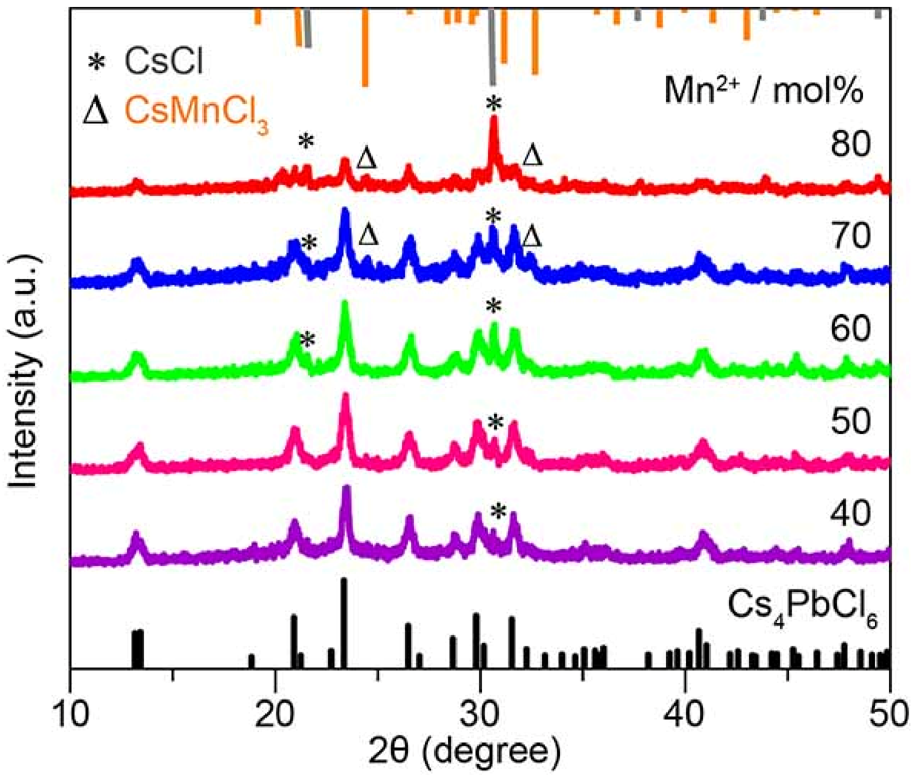

Figure S1. XRD patterns of Cs_4PbCl_6:Mn^{2+} NCs with nominal Mn^{2+} doping concentrations varying from 40 mol% to 80 mol%. The bottom lines represent the standard XRD pattern of rhombohedral Cs_4PbCl_6 (JCPDS No. 76-1530), and the top lines represent the standard XRD patterns of CsCl (grey, JCPDS No. 05-0607) and $CsMnCl_3$ (orange, JCPDS No. 00-2525). Impurity phases of CsCl and $CsMnCl_3$ were observed when the nominal Mn^{2+} doping concentration was higher than 40 mol%, due to spinodal decomposition of the NCs.

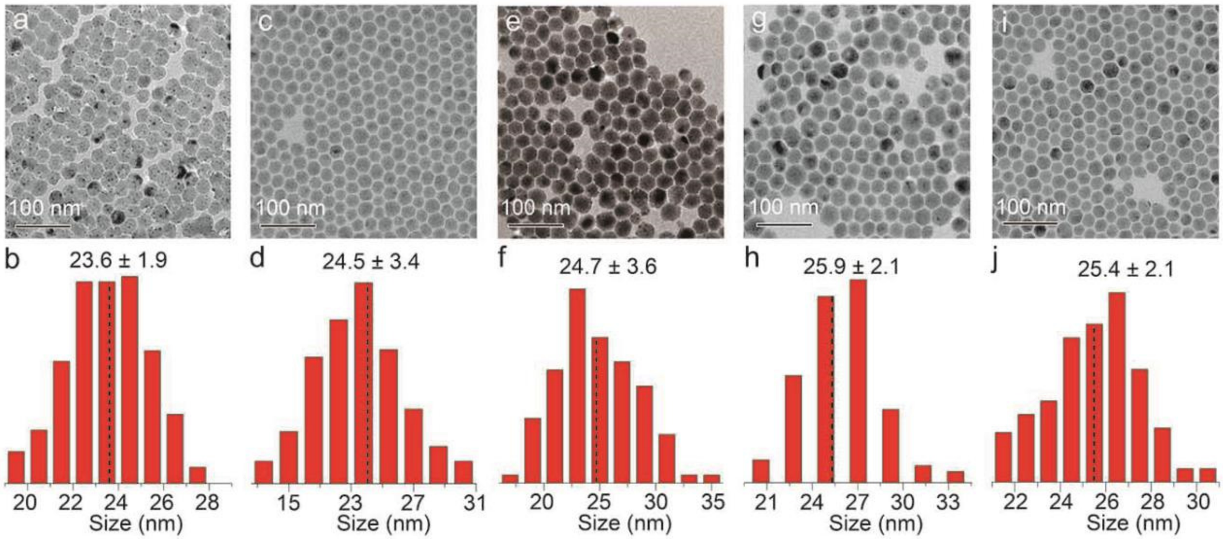

Figure S2. TEM images and size distributions of $Cs_4PbCl_6:Mn^{2+}$ NCs with Mn^{2+} doping concentrations of a,b) 0, c,d) 0.7, e,f) 2.1, g,h) 13.3, and i,j) 23.6 mol%. The size distributions of the NCs were obtained by calculating 200 particles in the TEM images.

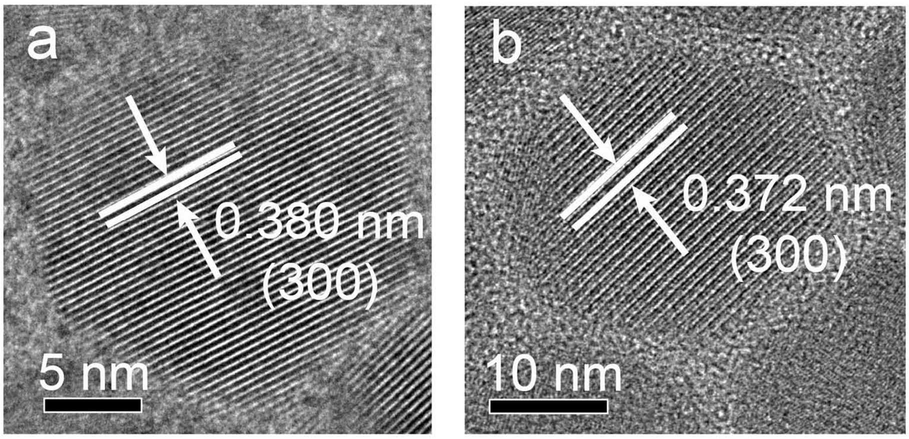

Figure S3. High-resolution TEM images for a) the undoped and b) 23.6 mol% Mn^{2+}-doped Cs_4PbCl_6 NCs. The interplanar distances of the (300) plane for the undoped and 23.6 mol% Mn^{2+}-doped Cs_4PbCl_6 NCs were determined to be 0.380 nm and 0.372 nm, respectively, confirming the lattice contraction of the NCs induced by Mn^{2+} doping.

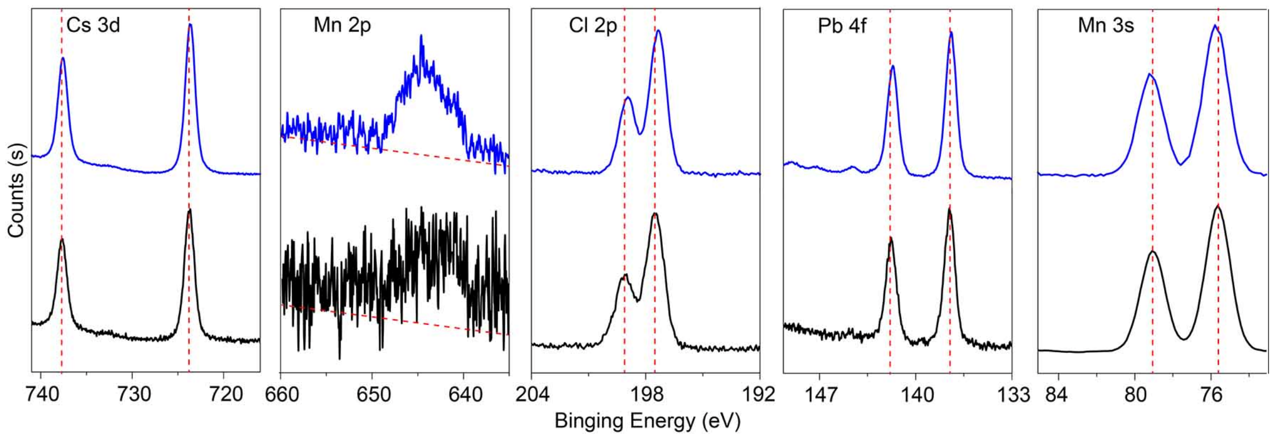

Figure S4. XPS spectra of Cs_4PbCl_6:Mn^{2+} NCs with Mn^{2+} doping concentrations of 0.7 mol% (black) and 23.6 mol% (blue). The spectra are shown over the energy regions typical for Cs 3d, Pb 4f, Mn 2p, Mn 3s, and Cl 2p peaks. The two bimodal peaks of Cs 3d, Pb 4f, Mn 3s and Cl 2p were located at the binding energies of 737.6 and 723.7 eV, 142.9 and 138.1 eV, 77.0 and 74.7 eV, and 199 and 197.4 eV, respectively. The peak of Mn 2p was located at 645.4 eV.

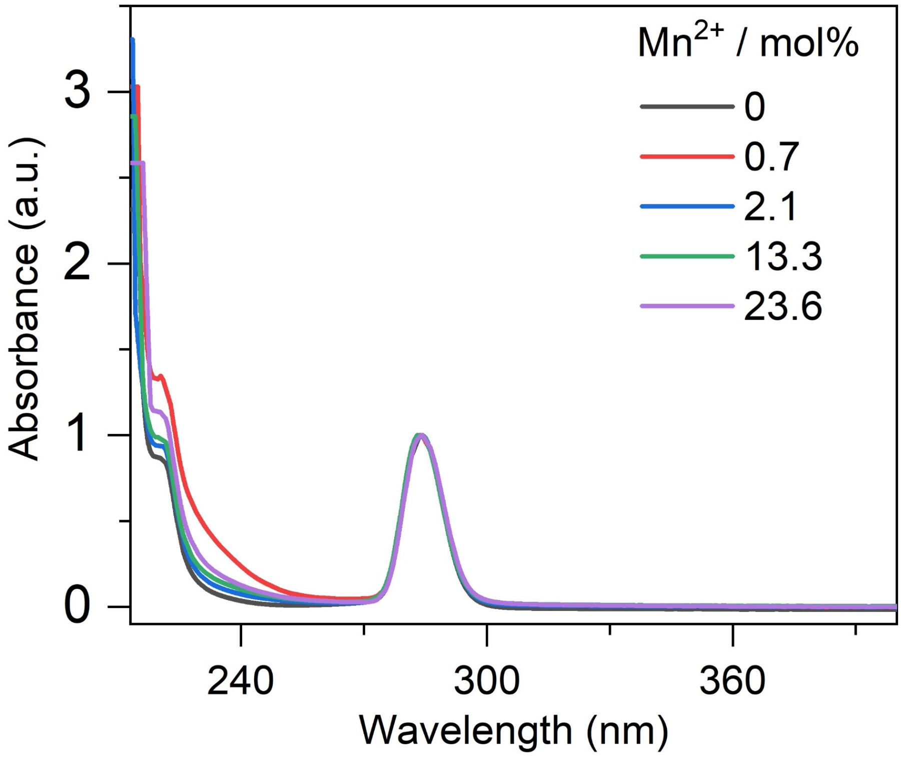

Figure S5. Optical absorption spectra of Cs_4PbCl_6:Mn^{2+} NCs with different Mn^{2+} doping concentrations, normalized at 284 nm. In addition to the localized exciton absorption band (A band) at 284 nm, all the NCs exhibited continuum absorption bands at higher energies, with intensities much stronger than that of the exciton absorption band. The continuum absorption band at 220 nm (B band) was ascribed to the vibration-induced $^1S_0 \rightarrow {}^3P_2$ transition of Pb^{2+}, and the bands at higher energies (C band) out of the instrument response were assigned to the dipole-allowed $^1S_0 \rightarrow {}^1P_1$ transition of Pb^{2+} in Cs_4PbCl_6 NCs

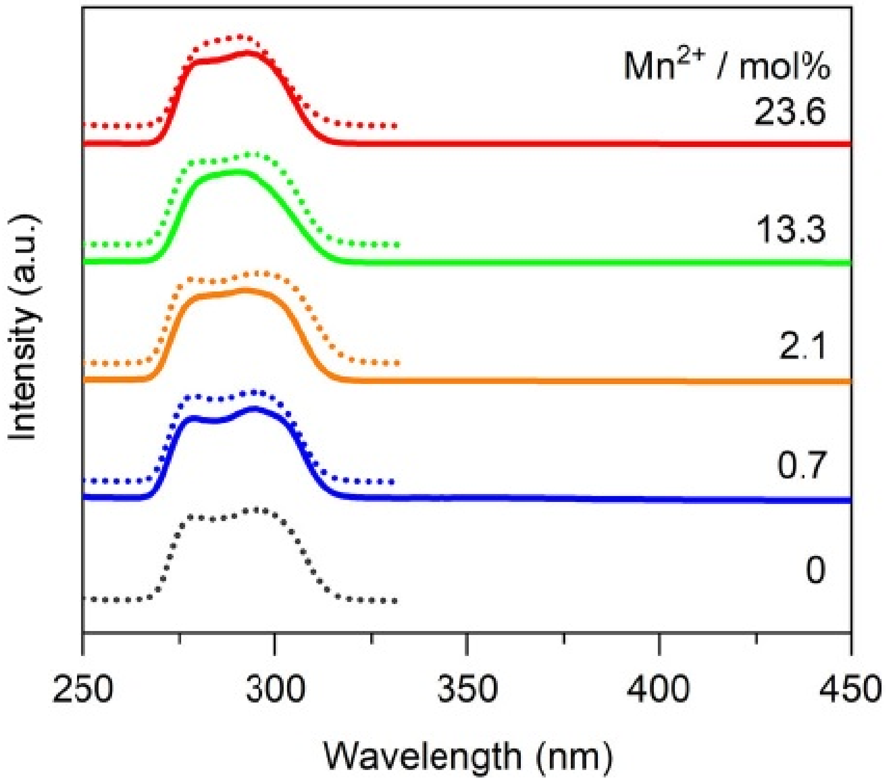

Figure S6. PL excitation spectra of Cs_4PbCl_6:Mn^{2+} NCs with different Mn^{2+} doping concentrations by monitoring the Pb^{2+} (dot line) and Mn^{2+} (solid line) emissions at 358 and 617 nm, respectively. All the NCs exhibited a similar broad excitation band around 289 nm for both Pb^{2+} and Mn^{2+} emissions, ascribed to the $^1S_0 \rightarrow {}^3P_1$ transition of Pb^{2+} in Cs_4PbCl_6 NCs. This provides a solid evidence for the efficient energy transfer from Pb^{2+} to Mn^{2+} in Cs_4PbCl_6:Mn^{2+} NCs. Note that no excitation band in the spectral range from 350 to 450 nm was detected by monitoring the Mn^{2+} emission, indicating that the NCs were free of $CsPbCl_3$:Mn^{2+} impurity.

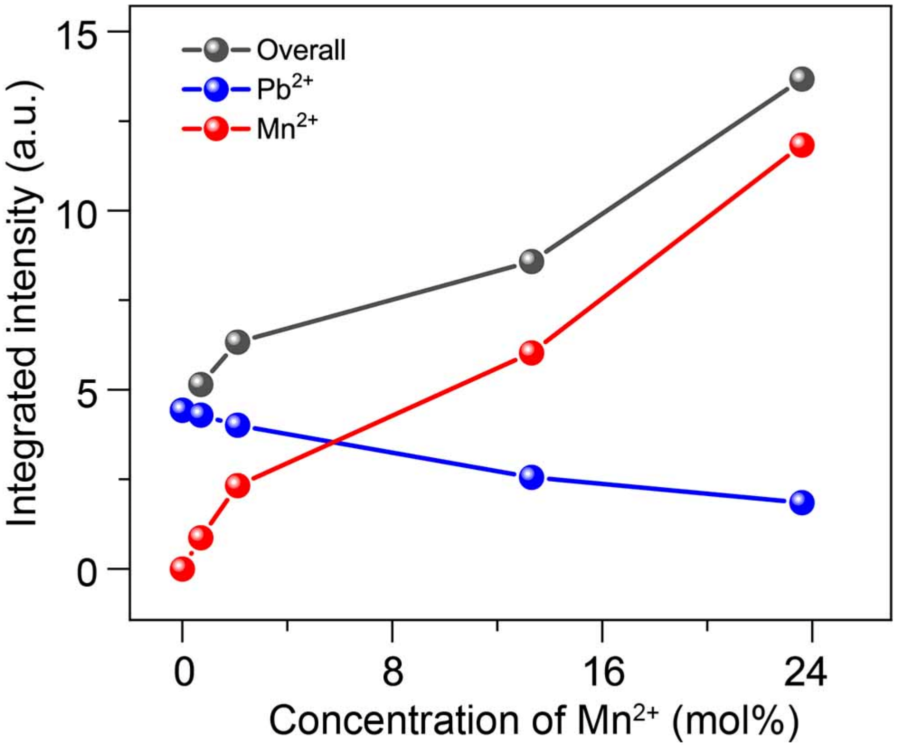

Figure S7. Integrated PL intensities of Pb^{2+} and Mn^{2+} in Cs_4PbCl_6:Mn^{2+} NCs and the overall PL intensity of the NCs as a function of Mn^{2+} doping concentrations, showing a gradual increase in Mn^{2+} emission at the expense of the Pb^{2+} emission with increasing the Mn^{2+} concentration. The overall PL intensity of the NCs was enhanced by factors of 1.2, 1.5, 1.9, and 3.1 as the Mn^{2+} concentration increased from 0 to 0.7, 2.1, 13.3, and 23.6 mol%, respectively, as a merit of efficient energy transfer from Pb^{2+} to Mn^{2+} which alleviated the energy migration through Pb^{2+} sublattice to the intrinsic or surface defects of the NCs.

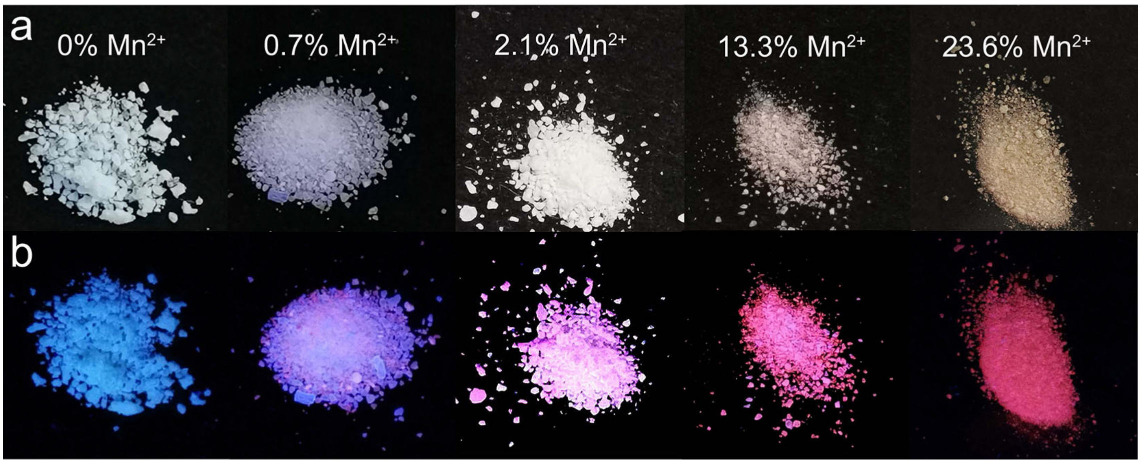

Figure S8. Photographs of Cs_4PbCl_6:Mn^{2+} NC powders with different Mn^{2+} doping concentrations on the glass slides under a) ambient and b) UV light. Under UV lamp irradiation at 304 nm, the NC powders exhibited bright PL with their emission colors changed from blue to pink with increasing the Mn^{2+} doping concentration, confirming the high PLQYs of Cs_4PbCl_6:Mn^{2+} NCs in solid form.

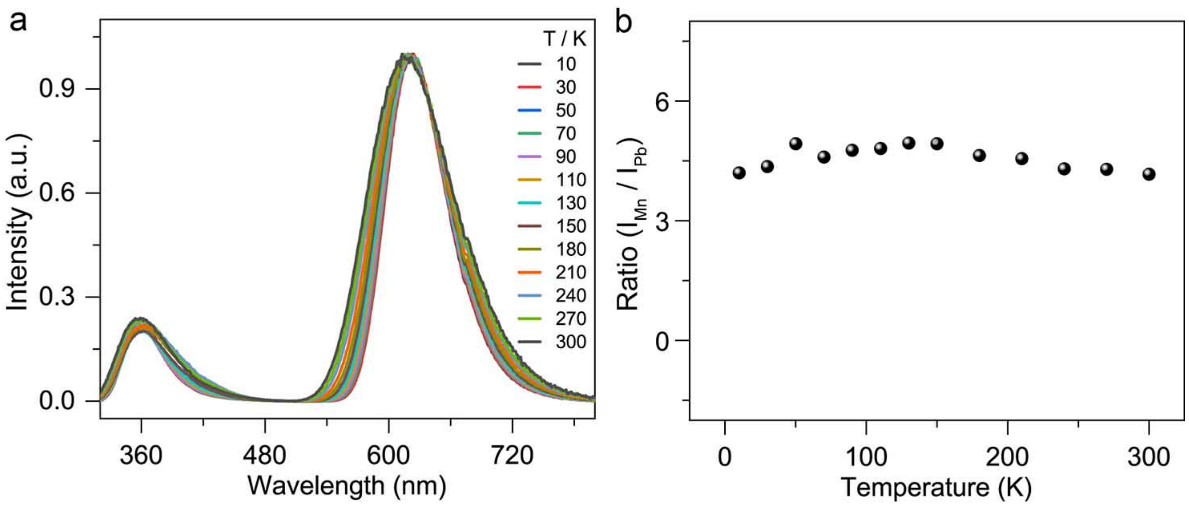

Figure S9. a) Normalized temperature-dependent PL emission spectra (λ_{ex} = 289 nm) of Cs_4PbCl_6:23.6% Mn^{2+} NCs. b) PL intensity ratio of Mn^{2+} to Pb^{2+} in Cs_4PbCl_6:23.6% Mn^{2+} NCs as a function of temperature, showing a nearly unchanged PL intensity ratio of Mn^{2+} to Pb^{2+} with the temperature decrease.

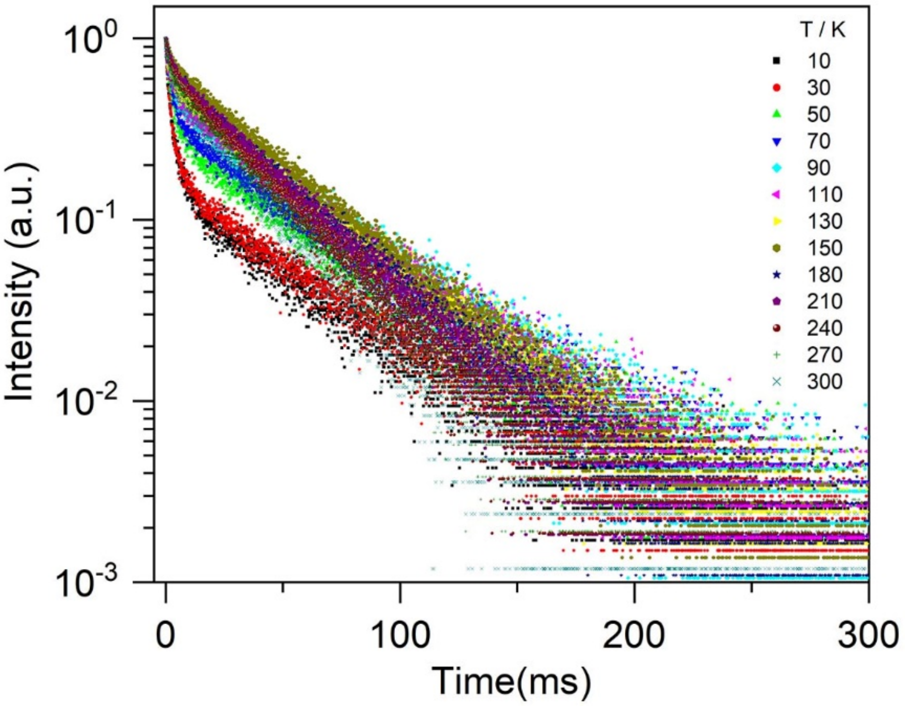

Figure S10. Temperature-dependent PL decay curves (λ_{ex} = 289 nm) of Cs_4PbCl_6:23.6% Mn^{2+} NCs by monitoring the Mn^{2+} emission at its maximum around 617 nm. All the decay curves were fitted to a biexponential function $\mathrm{I(t)} = A_1e^{-t/\tau_1} + A_2e^{-t/\tau_2}$ consisting of a fast decay component with a time constant τ_1 around 1~2 ms and a slow decay component with a time constant τ_2 in tens of ms scale. The average lifetimes were determined by the expression $\tau_{ave} = (A_1\tau_1^2 + A_2\tau_2^2)/(A_1\tau_1 + A_2\tau_2)$. The time constants and the amplitudes of the decay components were summarized in Table S4.

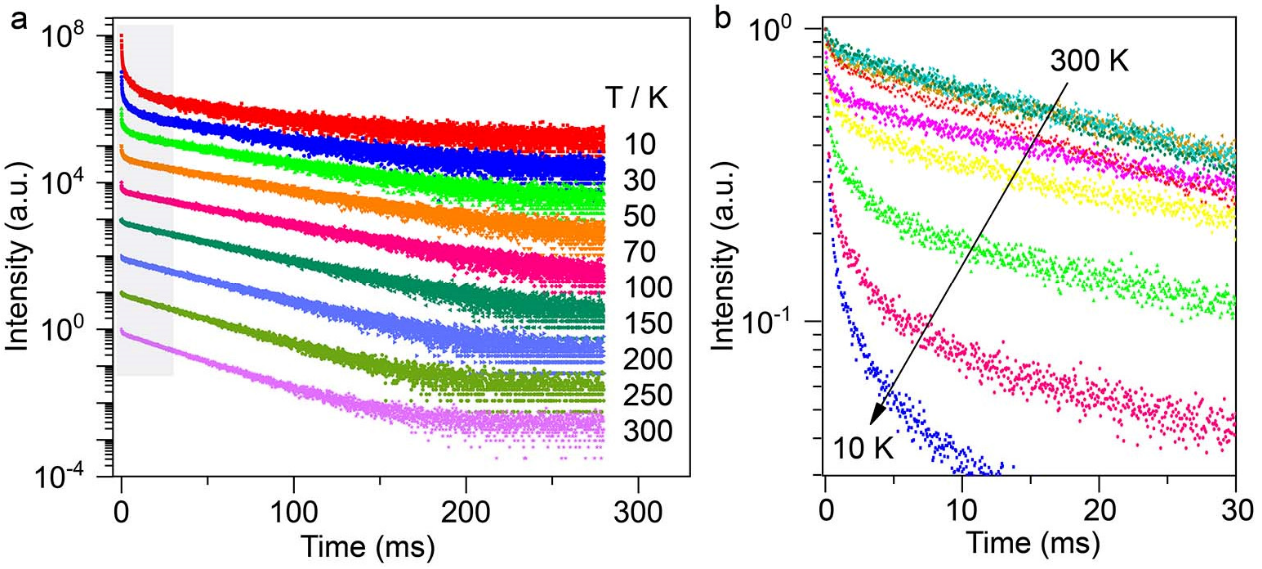

Figure S11. a) Temperature-dependent PL decay curves (λ_{ex} = 289 nm) of Cs_4PbCl_6:0.7% Mn^{2+} NCs by monitoring the Mn^{2+} emission at its maximum around 607 nm. The decay curves were stacked up to make each of them more visible. The initial fast decay portion marked by shadow was enlarged in b), showing a remarkable increase in the amplitude of the fast Mn^{2+}-PL decay with the temperature decrease.

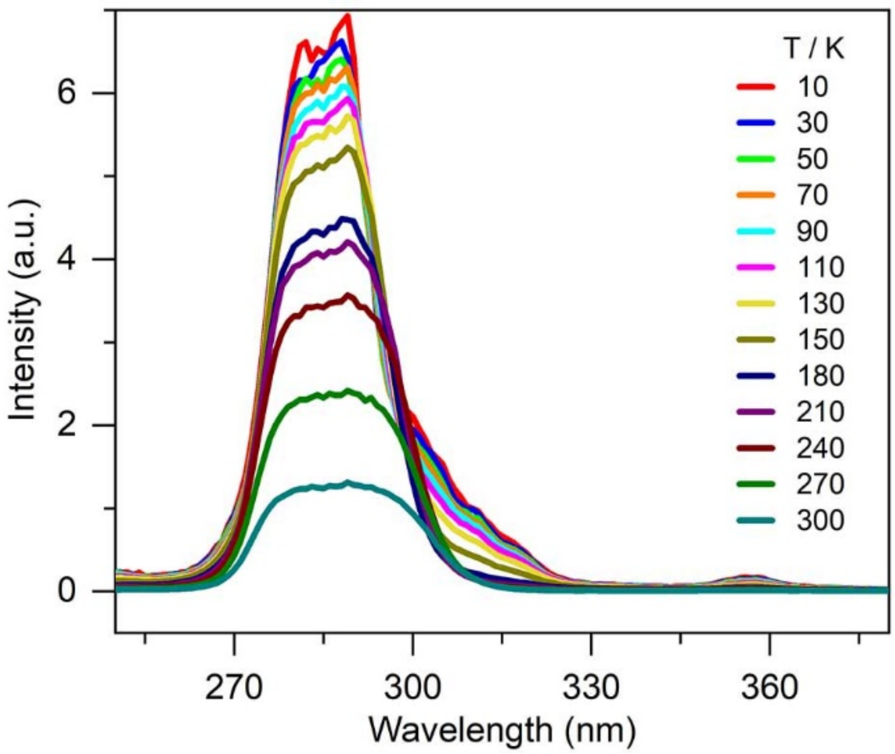

Figure S12. Temperature-dependent PL excitation spectra of Cs_4PbCl_6:23.6% Mn^{2+} NCs by monitoring the Mn^{2+} emission around 617 nm. No excitation band related to the absorption of $CsPbCl_3$ was observed, confirming that the fast Mn^{2+}-PL decay was not from $CsPbCl_3$:Mn^{2+} impurity. The weak excitation band around 356 nm at temperatures below 150 K is most probably related to the absorption of Mn^{2+} stemmed from strong Mn−Mn dipole−dipole coupling interactions which lead to an increase in the transition probability of Mn^{2+}.

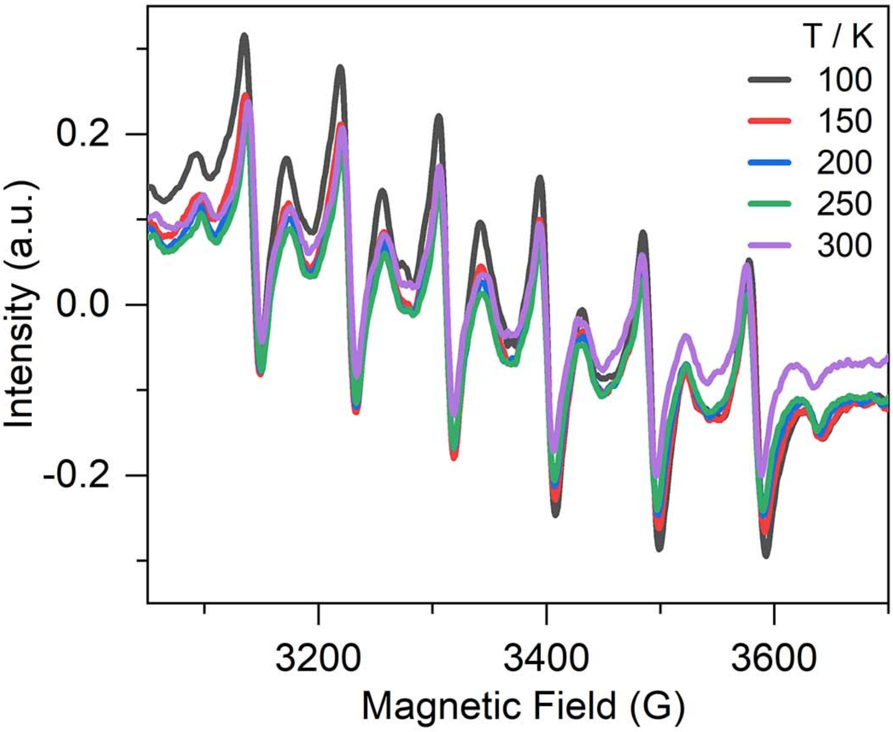

Figure S13. X-band EPR spectra of Mn^{2+} in Cs_4PbCl_6:0.7% Mn^{2+} NCs measured at different temperatures, showing a gradual broadening of the EPR bands of Mn^{2+} as the temperature decreased from 300 to 100 K.
